# Dr. Albert E. Stafford

**Published:** 1918-08

**Authors:** 


**Dr. Albert E. Stafford** of Whitesbore, N. Y. died recently, age 41. He graduated from the University of Syracuse.

